# Hyperbaric oxygen therapy for necrotizing fasciitis: a narrative review of mechanisms, clinical evidence, controversies, and practical considerations

**DOI:** 10.3389/fcimb.2026.1842036

**Published:** 2026-07-17

**Authors:** Yansong Xu, Chunyan Huang

**Affiliations:** Emergency Department, The First Affiliated Hospital of Guangxi Medical University, Nanning, China

**Keywords:** hyperbaric oxygen therapy, mechanisms of action, multidisciplinary management, narrative review, necrotizing fasciitis

## Abstract

Necrotizing fasciitis (NF) is a life-threatening infection requiring urgent, multidisciplinary care. This review examines the role of adjunctive hyperbaric oxygen therapy (HBOT), including its mechanisms and clinical evidence. HBOT increases tissue oxygen tension, thereby enhancing neutrophil bactericidal activity, inhibiting anaerobic bacteria, reducing edema, modulating inflammation, and promoting angiogenesis and wound healing. Although large-scale randomized trials are lacking, meta-analyses and cohort studies associate HBOT with reduced mortality and amputation rates, shorter hospital stays, and more efficient resource use, particularly in severe cases such as Fournier’s gangrene. However, its application remains controversial due to study limitations, cost, and potential risks. This narrative review does not provide a quantitative synthesis of effect estimates, and conclusions should be interpreted with awareness of the predominantly observational nature of the available evidence. In conclusion, current evidence suggests that adjunctive HBOT may be associated with improved outcomes in NF, especially when initiated early after surgical debridement. Nevertheless, the absence of large-scale randomized controlled trials, significant heterogeneity across studies, and concerns regarding cost and logistics preclude definitive recommendations. The therapeutic role of HBOT likely depends on optimal patient selection and seamless integration within a multidisciplinary framework. Future prospective studies are essential to clarify its efficacy, cost-effectiveness, and precise indications.

## 1 Introduction

Necrotizing soft tissue infections (NSTIs) carry a persistently high mortality rate of 20%–40% despite standard care, which includes prompt surgical debridement, broad-spectrum antibiotics, and intensive supportive therapy (Abbasi et al., 2025; Zhou et al., 2025). This persistently high mortality underscores a critical therapeutic gap: the unchecked “second hit” of systemic inflammation, microvascular dysfunction, and tissue hypoxia that can lead to organ failure (Perl et al., 2025; Yeh et al., 2026).

Hyperbaric oxygen therapy (HBOT)—an intervention that delivers 100% oxygen at supra-atmospheric pressure—has been utilized worldwide as an adjunctive treatment for NSTI for decades, despite the absence of large-scale randomized controlled trials. By reversing tissue hypoxia and modulating immune responses, HBOT offers a biological rationale for improving outcomes in these severe infections (Fu et al., 2022; Mladenov et al., 2022). However, its clinical role remains controversial. While some studies report associations between HBOT and reduced mortality or amputation rates (Huang et al., 2023; Hyldegaard et al., 2025), others have failed to demonstrate significant independent survival benefits after adjusting for confounders such as treatment timing and patient comorbidities (Mladenov et al., 2022; Ortega et al., 2021). These contradictory findings have led to ongoing debate regarding the efficacy of HBOT in NSTI.

Several factors may explain the heterogeneity in reported outcomes. Differences in patient selection, timing of HBOT initiation, treatment protocols (pressure, duration, frequency), and the severity of illness across studies likely contribute to the variability in observed effects. Importantly, most available evidence derives from observational studies with inherent selection biases, and large-scale randomized controlled trials (RCTs) are notably absent (Huang et al., 2023). Consequently, it remains unclear whether the inconsistent results reflect true variation in treatment efficacy, differences in study populations, or methodological limitations.

Current evidence does suggest that certain patient subgroups—particularly those with septic shock or higher illness severity scores—may derive more pronounced benefit from adjunctive HBOT (Hyldegaard et al., 2025; Yang et al., 2023). However, the concept of “extensive wounds” as a selection criterion remains poorly defined and lacks standardized measurement. Furthermore, translating these observations into clinical practice is challenging, as precise patient selection based on available evidence is often neither straightforward nor consistently feasible in real-world emergency settings. Given these uncertainties, this review aims to provide a balanced synthesis of current knowledge on HBOT for NSTI. We examine the proposed mechanisms of action, critically evaluate the clinical evidence—including its strengths and limitations—and discuss practical considerations for implementation. Rather than advocating for a fixed protocol or definitive framework, we seek to clarify the current state of evidence, identify knowledge gaps, and offer guidance for clinicians navigating the complex decision of whether and when to employ HBOT in patients with necrotizing fasciitis.

## 2 Literature search strategy

This narrative review was not conducted as a formal systematic review; however, to ensure a comprehensive and reproducible literature synthesis, we performed a structured search of the PubMed, Web of Science, and Cochrane Library databases. The search strategy combined keywords related to the intervention (‘hyperbaric oxygen therapy’,’HBOT’,’hyperbaric oxygenation’) and the condition (‘necrotizing fasciitis’,’necrotizing soft tissue infection’,’Fournier’s gangrene’). The search covered the period from database inception to March 2025. We primarily included clinical studies (randomized controlled trials, prospective/retrospective cohort studies, case-control studies, and case series) and relevant meta-analyses published in English. Exclusion criteria included non-peer-reviewed articles, conference abstracts, editorials, and animal or *in vitro* studies that lacked direct clinical correlation. Reference lists of included articles were also manually screened for additional relevant studies. No formal quality assessment tool was applied to the included studies, and this review does not aim to provide a quantitative synthesis of effect estimates. Instead, we synthesize the available literature to discuss mechanisms, clinical evidence, controversies, and practical considerations regarding HBOT for NF. Two authors (YX and CH) independently screened titles and abstracts of all retrieved records. Full texts of potentially eligible studies were then assessed against the eligibility criteria. Disagreements were resolved by discussion or, if necessary, by consultation with a third reviewer.

## 3 Theoretical basis and mechanism of HBOT treatment for NF

**Figure 1:**
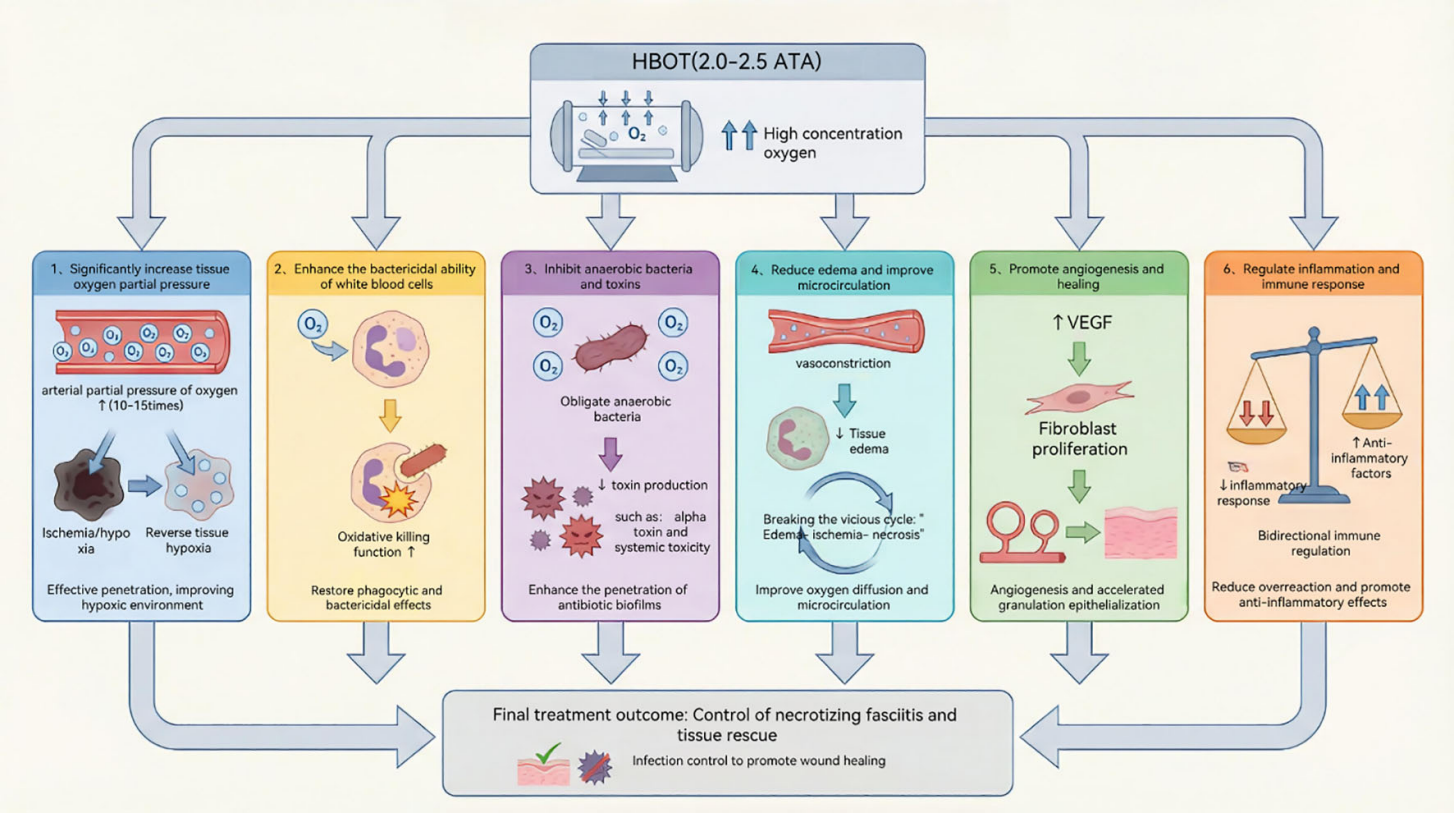
Schematic overview of the multi-tiered mechanisms of hyperbaric oxygen therapy in infectious disease.

The therapeutic efficacy of HBOT in necrotizing fasciitis stems from a series of synergistic, multi-tiered pathophysiological mechanisms triggered by the hyperoxic environment it creates. These mechanisms range from acute physicochemical effects to complex cellular signaling modulation and immunoregulation, collectively forming a multidimensional and interconnected biological network (**Figure 1**).

### 3.1 Significant elevation of tissue oxygen tension

HBOT significantly increases tissue oxygen partial pressure by administering 100% oxygen to patients at 2–3 atmospheres absolute. According to Henry’s law, this dramatically enhances the physical dissolution of oxygen in plasma—reaching levels 10–20 times higher than at normobaric conditions—thereby directly and substantially raising tissue oxygen tension (He et al., 2023; Huang et al., 2023; Yang et al., 2023). This core effect reverses tissue hypoxia in the central zone of infection. It operates through two primary pathways relevant to NF: first, by providing a critical substrate that enhances the oxidative bactericidal function of neutrophils against invading pathogens; and second, by creating a tissue environment hostile to obligate anaerobic bacteria, thereby synergistically aiding infection control.

### 3.2 Antimicrobial mechanisms: beyond direct bacterial killing

The antimicrobial effects of HBOT extend beyond simple oxygen-mediated bacterial inhibition, encompassing enhancement of host immune function, synergy with antibiotics, and disruption of biofilm structures.

Neutrophil-Mediated Killing: Hypoxia profoundly impairs the oxidative killing capacity of neutrophils. HBOT directly reverses this dysfunction by providing the essential substrate—oxygen—required for robust reactive oxygen species (ROS) generation via NADPH oxidase and the mitochondrial electron transport chain (He et al., 2023). By dramatically elevating tissue oxygen tension, HBOT not only restores but potentiates neutrophil phagocytic and bactericidal activity, particularly against aerobic pathogens (Fu et al., 2022; He et al., 2023).

Synergistic Effects with Antibiotics: HBOT enhances the efficacy of several antibiotics commonly used in NSTI. In a murine model of chronic wound infection, Laulund et al. (2023a) demonstrated that adjunctive HBOT significantly augmented the bactericidal effect of ciprofloxacin against *Pseudomonas aeruginosa* biofilms, achieving bacterial reduction superior to either monotherapy (Laulund et al., 2023a). Similar synergistic effects have been reported with aminoglycosides in hyperoxia tissue environments, where conventional antibiotic efficacy is often compromised (Bombaywala et al., 2024).

Disruption of Biofilm Microenvironment: HBOT directly targets biofilm structures. Laulund et al. (2023b) showed that HBOT disrupts the biofilm micro-compartment phenomenon in *P. aeruginosa*—a mechanism by which bacteria within biofilms create localized hypoxic niches that shield them from immune clearance and antibiotics (Laulund et al., 2023b). By increasing oxygen penetration into biofilm matrices, HBOT renders sessile bacteria more susceptible to both neutrophil-mediated killing and antimicrobial agents.

Effects on Anaerobic Bacteria: In contemporary clinical practice, obligate anaerobes — including *Clostridium perfringens* — account for a minority of NSTI cases, with most infections being polymicrobial involving both aerobes and anaerobes (Jia et al., 2025; Kiu et al., 2023). HBOT suppresses anaerobic bacteria by elevating tissue oxygen tension, creating an environment hostile to their oxygen-sensitive metabolism (Huang et al., 2023; Laulund et al., 2023a; Mladenov et al., 2022). For toxigenic anaerobes such as *C. perfringens*, hyperbaric oxygen has been shown to inhibit the production of alpha toxin, a major virulence factor, thereby potentially reducing tissue destruction and systemic toxicity (Van Unnika, 1965). However, the clinical relevance of this mechanism should be considered within the broader context of HBOT’s multifaceted antimicrobial effects, which extend to aerobes and biofilms as described above.

### 3.3 Anti-inflammatory and anti-edema effects

**Figure 2:**
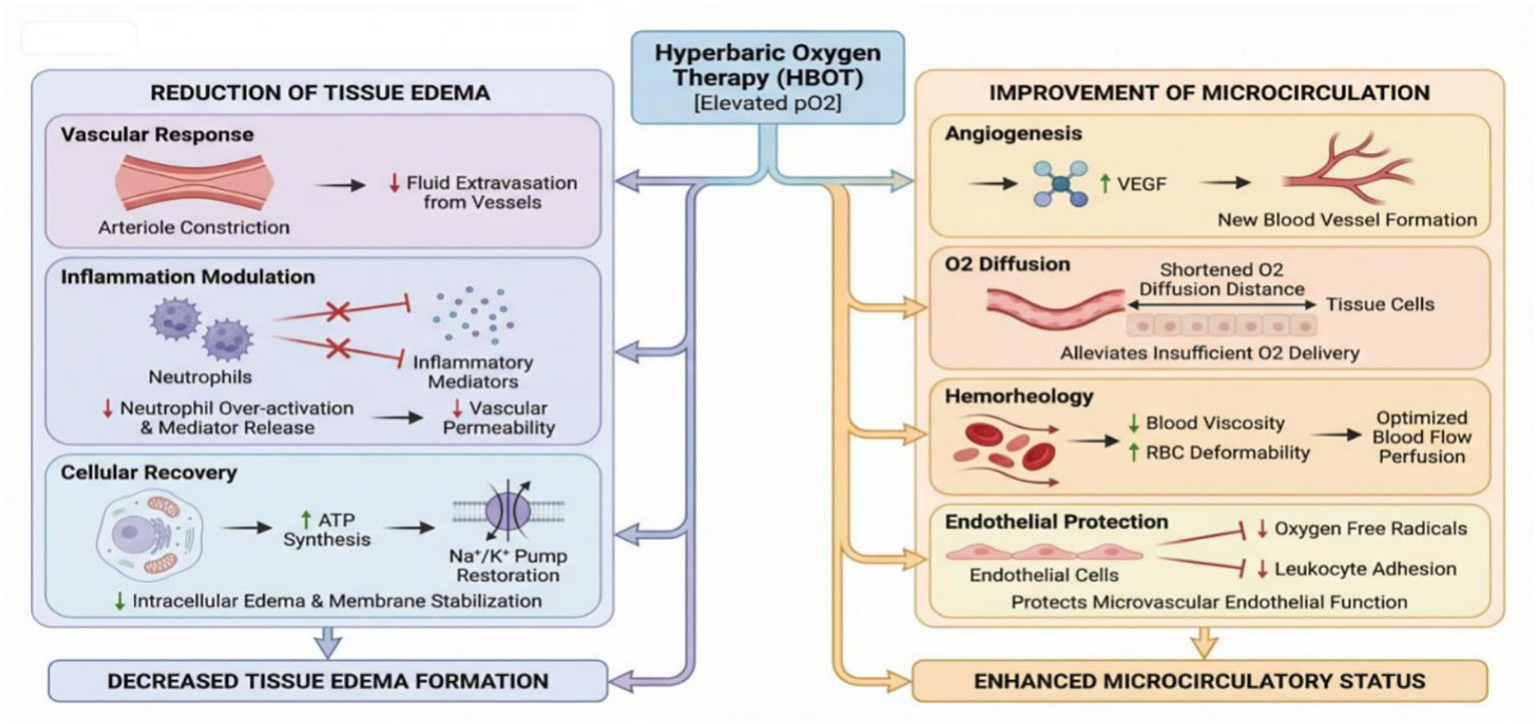
Dual effects of hyperbaric oxygen on tissue edema reduction and microcirculatory improvement.

A pivotal benefit of HBOT in NSTI is its dual capacity to reduce tissue edema while concurrently modulating the inflammatory response—processes essential for salvaging ischemic tissue (**Figure 2**).

Edema reduction: HBOT counteracts edema through convergent mechanisms. It induces arteriolar constriction, limiting fluid extravasation—a critical effect in ischemia-reperfusion injury (He et al., 2025). Furthermore, HBOT attenuates ischemia-reperfusion injury by modulating leukocyte-endothelial interactions at the molecular level. Hyperoxia downregulates the expression of β-2 integrins (CD11/CD18) on the surface of activated neutrophils, thereby reducing their adhesive capacity (Larson et al., 2000). Concurrently, it dampens neutrophil-driven inflammation, thereby decreasing vascular permeability (Mallat et al., 2022). Furthermore, by restoring cellular oxygen levels and ATP production, HBOT reactivates the sodium-potassium pump, correcting intracellular edema and stabilizing membrane integrity (Yang et al., 2023).

Inflammation modulation: HBOT exerts broad-spectrum immunomodulatory effects. Controlled ROS generation acts as signaling molecules that recalibrate dysregulated inflammation, attenuating the destructive cytokine storm while preserving defensive capacity (He et al., 2025). Clinical studies have demonstrated that HBOT significantly lowers circulating pro-inflammatory cytokines, including IL-1β, IL-6, and TNF-α, in NSTI patients (Kiu et al., 2023).

### 3.4 Microcirculatory restoration and angiogenesis

Complementing its anti-edema effects, HBOT actively may be associated with improvement in tissue perfusion and promotes long-term vascular repair.

Microcirculatory enhancement: By vastly increasing oxygen diffusion gradient, HBOT functionally shortens the distance oxygen must travel from capillaries to hypoxic cells, overcoming diffusion-limited oxygen delivery. HBOT also optimizes blood flow by reducing viscosity and improving erythrocyte deformability (Yang et al., 2023), while its antioxidant and anti-adhesive effects protect the microvascular endothelium from further ischemia-reperfusion damage (Groborz et al., 2025).

Angiogenesis and tissue repair: Beyond its acute anti-infective and anti-edema roles, HBOT also promotes long-term wound healing through mechanisms that operate over days to weeks. It is important to emphasize that the following repair mechanisms—angiogenesis, fibroblast activation, and matrix remodeling—contribute primarily to wound healing in the subacute and chronic phases of care, rather than to acute survival benefit in the initial days of NSTI management. These chronic repair mechanisms do not explain the acute mortality reduction observed in clinical studies where HBOT was initiated early after surgical debridement. The master regulator of this shift is Hypoxia-Inducible Factor-1α (HIF-1α), which is stabilized under low oxygen conditions and drives a maladaptive, pro-inflammatory state. HBOT, by dramatically raising tissue oxygen concentration, promotes the hydroxylation and proteasomal degradation of HIF-1α, thereby switching off this pathological program (Arienti et al., 2021; Dejonckheere et al., 2026).

This oxygen-sensing switch initiates a coordinated repair cascade: (1)Angiogenesis: The alleviation of hypoxia stimulates the release of potent pro-angiogenic factors such as VEGF and SDF-1, driving endothelial cell proliferation and the formation of new, functional blood vessels to restore perfusion (Dejonckheere et al., 2026; Fu et al., 2022; Liu et al., 2026). (2)Matrix Deposition & Remodeling: Direct elevation of oxygen tension activates fibroblasts, enhancing collagen synthesis and promoting organized extracellular matrix deposition critical for tissue strength (Dejonckheere et al., 2026; Fu et al., 2022; Liu et al., 2026).(3)Anti-Inflammatory & Pro-Energetic Shift: HBOT downregulates key inflammatory mediators like TNF-α, dampening chronic inflammation. Concurrently, it enhances mitochondrial oxidative phosphorylation, providing the necessary bioenergetic support for the high metabolic demands of keratinocytes, fibroblasts, and other repair cells (Fu et al., 2022; Liu et al., 2026).

## 4 Clinical efficacy evidence: data and controversy

The current evidence supporting HBOT for necrotizing soft tissue infections (NSTIs) primarily derives from retrospective studies and a limited number of prospective investigations. In the largest prospective cohort to date, Hyldegaard et al. (2025) reported that HBOT was associated with a significantly lower 30-day mortality (7% vs. 43%) (**Table 1**), with a notable absence of large-scale randomized controlled trials (RCTs). Despite the consistency of many findings, it is imperative to critically appraise the evidence base. The vast majority of studies cited in this review—including those summarized in **Table 1**—are retrospective and observational in design. These methodologies are inherently susceptible to several biases. First, selection bias may occur if patients receiving HBOT differ systematically from those who do not (e.g., healthier patients may be more likely to be referred for HBOT, or conversely, HBOT may be reserved as a ‘last resort’ for the most critically ill). Second, confounding by indication is a major concern, as the decision to initiate HBOT is often influenced by disease severity, which itself is a strong predictor of mortality. Third, few studies adequately control for key confounders such as time from symptom onset to first surgical debridement, specific antibiotic regimens, or the intensity of critical care support.

**Table 1:** Core evidence table for hyperbaric oxygen therapy in necrotizing fasciitis/severe soft tissue infections (2010-2025).

Author (year)	Study type	Patient number (HBOT/non-HBOT)	Key findings	Risk of bias	Level of evidence & limitations
Prospective Studies					
(Hyldegaard et al., 2025)	Prospective, Observational, Multicenter Cohort	409 (329/80)	HBO_2_ treatment was associated with significantly lower 30-day (7% vs 43%) and 90-day (11% vs 46%) all-cause mortality compared to non-HBO_2_ patients.	Moderate	Level 2 (prospective cohort) Limitations: Non-randomized; selection bias; protocol variability across centers
(Hedetoft et al., 2021)	Prospective Observational Study	80 patients (all received HBOT)	HBOT increased sICAM-1 levels; low baseline sICAM-1 predicted 90-day mortality	Serious	Level 2 (prospective cohort) Limitations: No control group; small sample size
Retrospective Studies					
(Shaw et al., 2014)	Retrospective, multi-institutional with PSM	1,583 (117/1,466)	HBOT associated with significant survival benefit (OR for death without HBOT = 10.6); benefit strongest in sickest patients	Moderate	Level 3 (PSM cohort) Limitations: Administrative data (ICD-9); no clinical details on HBOT protocol
(Soh et al., 2012)	Nationwide inpatient sample study with PSM	45,913 (405/45,508 after matching)	HBOT associated with lower mortality (4.5% vs 9.4%; aOR=0.49) despite longer stays and higher costs	Moderate	Level 3 (PSM cohort) Limitations: Administrative data; no post-discharge follow-up; potential coding errors
(Shishido et al., 2025)	Retrospective cohort (single-center)	253 (143/110)	90-day mortality lower in HBOT group (5.8% vs 15.4%, p=0.015); benefit strongest in APACHE II ≥18 and wounds ≥450 cm²	Serious	Level 3 (retrospective cohort) Limitations: Single-center; selection bias; APACHE II not recorded at initial presentation
(Toppen et al., 2024)	Retrospective, nationwide cohort (NIS database) with PSM	4240 (2120/2120 after matching)	HBOT associated with lower in-hospital mortality (OR 0.63, 95% CI 0.47-0.86); higher total hospital costs but shorter length of stay; benefit consistent across sensitivity analyses	Moderate	Level 3 (PSM cohort) Limitations: no clinical details on disease severity (e.g., lactate, APACHE); potential coding errors; no post-discharge follow-up
(Mladenov et al., 2022)	Retrospective cohort (single-center)	187 (83/98)	HBOT group had more severe disease but similar survival; ineligibility for HBOT despite indication predicted mortality (OR = 8.59)	Serious	Level 3 (retrospective cohort) Limitations: Single-center; undocumented comorbidity severity; selection bias
(Creta et al., 2020)	Multi-institutional Observational Study	161 (72/89)	HBOT associated with lower mortality (19.4% vs 36.0%, p=0.01); independent predictor (OR = 0.074) in multivariate analysis	Serious	Level 3 (retrospective cohort) Limitations: Baseline severity imbalance; no standardized HBOT protocol
(Devaney et al., 2015)	Retrospective cohort (single-center)	341 (275/66)	Independent mortality predictors included HBOT, increased age, and immunosuppression	Serious	Level 3 (retrospective cohort) Limitations: Single-center; retrospective design
(Massey et al., 2012)	Retrospective cohort (single-center)	80 (32/48)	No significant difference in mortality or amputation; HBOT group had more debridement and higher perineal involvement	Serious	Level 3 (retrospective cohort) Limitations: Small sample; underpowered; no standardized HBOT protocol
(Kariksiz and Ates, 2025)	Retrospective cohort (single-center)	50 (HBOT in debridement group only)	Mortality 20%, amputation 60%; HBOT showed no significant effect on mortality or amputation	Serious	Level 3 (retrospective cohort) Limitations: Small sample; HBOT not randomized; no direct HBOT vs non-HBOT comparison
(Korambayil et al., 2024)	Retrospective comparative	24 (12/12)	HBOT with single-stage reconstruction had shorter hospital stay (p<0.05); no graft failure in either group	Serious	Level 3 (retrospective cohort) Limitations: Small sample; non-randomized allocation
(Feres et al., 2021)	Retrospective comparative (historical control)	198 (119/79)	Mortality lower in HBOT group (3.7% vs 28.8%, p<0.001)	Serious	Level 3 (retrospective cohort) Limitations: Historical control (different time periods); evolving medical practices
(Hassan et al., 2010)	Retrospective Comparative Study	48 (24/24)	Mortality lower in HBOT group (12.5% vs 29.2%), not statistically significant; fewer debridement in HBOT group	Serious	Level 3 (retrospective cohort) Limitations: Small sample; baseline severity may differ
Case Series					
(Steiner et al., 2018)	Retrospective case series (all HBOT)	91 (all received HBOT)	HBOT associated with improved survival without increased complications	Critical	Level 4 (case series) Limitations: No control group; selection bias
(Mehl et al., 2010)	Retrospective Case Series	26/24	Overall mortality similar to literature; no independent effect of HBOT evaluated	Critical	Level 4 (case series) Limitations: Non-comparative; unable to evaluate independent HBOT effect

Risk of bias was assessed qualitatively by the authors based on the following criteria: (1) adequacy of confounding control (e.g., propensity score matching, multivariable adjustment); (2) selection bias (e.g., single-center vs. multicenter; consecutive enrollment); (3) attrition bias (loss to follow-up); and (4) detection bias (objective vs. subjective outcomes). “Moderate” indicates some limitations but overall reliable; “Serious” indicates major limitations that may affect the estimate; “Critical” indicates the study is not interpretable due to severe flaws.

Level of evidence was assessed according to the Oxford Centre for Evidence-Based Medicine (OCEBM) 2011 criteria. For therapeutic studies: Level 2 = prospective cohort study with good follow-up; Level 3 = retrospective cohort study or case-control study; Level 4 = case series or historical control study; Level 5 = mechanism-based reasoning. All non-randomized studies are inherently limited by potential selection bias and confounding, which are noted in the limitation column.

In summary, while HBOT shows promise based on its theoretical mechanisms and some clinical observations, its clinical application is hindered by key controversies, the core issue being the lack of high-quality evidence. This evidence gap directly leads to clinical dilemmas, including the absence of clear patient selection criteria and uncertainty regarding the optimal therapeutic window. Furthermore, its resource-intensive nature raises questions about cost-effectiveness. Consequently, there is a consensus within the field on the urgent need for prospective studies, particularly well-designed RCTs, to definitively address these unresolved questions.

## 5 Clinical implementation of HBOT: protocol, safety, and collaborative strategies

As a critical adjunct within multidisciplinary NSTI management, the efficacy of HBOT depends on standardized protocols, stringent safety management, and seamless integration with cornerstone therapies. Its core mechanism of action lies in its ability to dramatically elevate tissue oxygen partial pressure, thereby correcting the hypoxic microenvironment within the infectious focus. This fundamental correction drives a cascade of therapeutic effects: enhancing leukocyte bactericidal function, inhibiting the growth of anaerobic bacteria, reducing tissue edema and inflammatory responses, and promoting neovascularization and tissue repair (Abri et al., 2022; Groborz et al., 2025).

### 5.1 Patient selection considerations

Based on the available evidence, certain patient subgroups have been reported to derive greater benefit from adjunctive HBOT. These include: (1) patients with **septic shock** as defined by Sepsis-3 criteria (vasopressor requirement and lactate >2 mmol/L) (Hedetoft et al., 2023; Hyldegaard et al., 2025); (2) patients with **high illness severity scores**, particularly APACHE II ≥18, elevated SAPS-2 scores (Hedetoft et al., 2023; Shishido et al., 2025); and (3) patients with **extensive wounds**, operationally defined as wound area ≥450 cm² after initial debridement (Shishido et al., 2025). However, it is critical to emphasize that these criteria are derived primarily from *post-hoc* subgroup analyses of retrospective studies. No prospective study has validated these thresholds for guiding HBOT decisions. Until such data emerge, clinical judgment regarding HBOT eligibility should integrate these preliminary criteria with individual patient characteristics, infection trajectory, and response to initial surgical and antibiotic therapy.

### 5.2 Standard treatment protocol

Clinical consensus emphasizes that HBOT should be initiated as early as possible after the first thorough surgical debridement to maximize its anti-infective and tissue-salvaging benefits, as delayed treatment may significantly reduce its efficacy (Zhou et al., 2025). According to the Undersea and Hyperbaric Medical Society (UHMS) Hyperbaric Medicine Indications Manual (15th edition, 2023), the recommended treatment protocol for NSTI consists of 90-minute sessions of 100% oxygen at an absolute pressure of 2.0 to 2.5 ATA, delivered twice daily for the first few days, and continued until there appears to be no further extension of necrosis in previously debrided areas and infection is controlled (Weaver, 2024). It is important to note that no randomized controlled trial has directly compared different HBOT protocols in patients with NF, and therefore, the optimal pressure, duration, and frequency remain undefined (see **Table 2**). Treatment Course: A complete course usually requires 5 to 10 consecutive days. The duration should be adjusted individually based on the patient’s clinical response, such as infection control status and wound granulation tissue growth.

**Table 2:** HBOT protocols uUsed in major clinical studies.

Study	Atmosphere absolute	Session duration (min)	Frequency
(Hyldegaard et al., 2025)	2.5	90	Twice daily
(Shishido et al., 2025)	2.0-2.4	90	Once or twice daily
(Hedetoft et al., 2021)	2.5	90	Twice daily
(Mladenov et al., 2022)	2.4-2.5	90-120	Not specified
(Creta et al., 2020)	2.0-2.5	90	Once or twice daily

### 5.3 Safety and adverse reaction management

A detailed medical assessment, including pulmonary and otorhinolaryngological examinations, is required before treatment. During the procedure, trained healthcare personnel must closely monitor the patient’s vital signs and subjective responses. HBOT is contraindicated in conditions such as untreated pneumothorax, certain pulmonary diseases (e.g., severe emphysema with carbon dioxide retention), and uncontrolled claustrophobia (Guenneugues et al., 2023). Common adverse reactions primarily include barotrauma to the middle ear or sinuses (which can be prevented by techniques such as the Valsalva maneuver), temporary myopia (usually reversible), and rare cases of oxygen toxicity (which can be classified into pulmonary and central nervous system types) (Groborz et al., 2025).

### 5.4 Multidisciplinary integrated treatment strategy

It is paramount to emphasize that HBOT is an adjunctive therapy and never a replacement for timely, extensive surgical debridement or appropriate antibiotic therapy. The ideal integrated treatment pathway consists of the following three phases: Acute Rescue Phase: The core interventions are emergency radical debridement, broad-spectrum intravenous antibiotics, and critical life support. HBOT should be initiated synchronously during this phase to help contain infection spread and salvage compromised tissue (Hyldegaard et al., 2025). Infection Control Phase: Antibiotic therapy is precisely adjusted according to intraoperative culture and sensitivity results. HBOT is continued to further eradicate residual infection, mitigate inflammation, and promote granulation tissue growth, thereby creating favorable conditions for subsequent repair (Laulund et al., 2023a; Laulund et al., 2023b). Recovery and Reconstruction Phase: The wound condition is assessed. Once infection is fully controlled, surgical procedures such as skin grafting or flap transfer are performed in a timely manner to achieve wound closure. During this phase, HBOT has been shown to potentially improve the survival rate of transplanted tissues and support overall healing, the outcomes can vary depending on individual factors and clinical conditions (Lin et al., 2023).

## 6 Future directions: toward evidence-based integration

To transcend current controversies and establish HBOT as a standard, precision adjunct in NF care, a coordinated strategy targeting evidence, delivery, and sustainability is imperative. The foremost need is for large-scale, multicenter, RCTs. These trials must be designed to answer critical, unresolved questions: Does early HBOT independently improve survival in specific high-risk subgroups (e.g., septic shock)? What is the optimal “therapeutic window” and dose? Robust RCT data are the essential foundation for all subsequent advancements.

A critical challenge in optimizing HBOT for NSTI is the lack of objective criteria to identify patients most likely to benefit. Recent research has investigated circulating biomarkers that may reflect disease severity and potentially inform treatment decisions. However, translation of biomarker data into clinical algorithms remains premature due to study limitations and unresolved questions regarding causality. **Table 3** summarizes prognostic biomarkers identified in NSTI patients, while **Table 4** presents studies examining biomarker modulation by HBOT. Despite these observations, several factors preclude the current use of biomarkers for guiding HBOT decisions: (1) most studies are observational with small sample sizes; (2) biomarker levels are influenced by timing of sampling and concomitant therapies; (3) causality between biomarker modulation and clinical benefit remains unclear; and (4) no prospective study has demonstrated that biomarker-guided HBOT may be associated with improved outcomes compared with standard care. Future research should prioritize validation of candidate biomarkers in prospective cohorts and development of clinically applicable algorithms that integrate clinical, microbiological, and biomarker data.

**Table 3:** Prognostic biomarkers in necrotizing soft tissue infections.

Biomarker category	Examples	Association with outcome	Key references
Pro-inflammatory cytokines	IL-6, IL-8, TNF-α	Higher levels correlate with disease severity and mortality	(Hansen et al., 2015); (Del et al., 2020)
Endothelial activation markers	sICAM-1, VCAM-1, thrombomodulin	Elevated levels associated with organ failure and poor prognosis	(Amalakuhan et al., 2016); (Hedetoft et al., 2021)
Acute phase proteins	CRP, procalcitonin	Elevated levels reflect infection severity; procalcitonin may help distinguish bacterial from non-bacterial causes	(Cherny et al., 2025)
Tissue hypoxia/metabolic markers	Lactate, pyruvate	High lactate predicts mortality and correlates with tissue hypoperfusion	(Hyldegaard et al., 2025); (Shishido et al., 2025)

All biomarkers listed are investigational. None have been prospectively validated for clinical decision-making in HBOT for NF. Reference ranges and clinically meaningful thresholds have not been established.

**Table 4:** Biomarker modulation by hyperbaric oxygen therapy in NSTI.

Biomarker	Observed effect of HBOT	Clinical correlation	Reference
sICAM-1	Significant increase, especially in septic shock patients	Low baseline levels predict 90-day mortality	(Hedetoft et al., 2021)
IL-1β, IL-6, TNF-α	Decreased circulating levels	Consistent with immunomodulatory effects	(Wu et al., 2018);
CRP	Variable; some studies report faster decline	May indicate more rapid infection control	(Feres et al., 2021)
Lactate	Faster clearance reported in HBOT-treated patients	Suggests improved tissue oxygenation and perfusion	(Hyldegaard et al., 2025)
VEGF, HIF-1α	Upregulated expression (preclinical)	Promotes angiogenesis and wound healing	(Li et al., 2021); (Tang et al., 2018)

The observed biomarker modulations are derived from research studies and do not imply clinical utility. None of these biomarkers are validated to guide HBOT treatment decisions (initiation, continuation, or termination).

## 7 Conclusion

HBOT exerts multiple biological effects that address key pathophysiological features of necrotizing fasciitis (NF), including reversal of tissue hypoxia, enhancement of neutrophil bactericidal activity, reduction of edema and inflammation, and promotion of angiogenesis and wound healing. Observational studies and meta−analyses have consistently reported associations between adjunctive HBOT and lower mortality and amputation rates, particularly in severely ill patients. However, the absence of large−scale randomized controlled trials precludes causal inference, and optimal treatment protocols (timing, pressure, duration, frequency) remain undefined. Reliable, prospectively validated criteria for patient selection are lacking, and the cost−effectiveness of HBOT relative to other interventions has not been established. Therefore, HBOT should be considered an adjunct—not a replacement—for timely surgical debridement and broad−spectrum antibiotics, with its use guided by local expertise, resource availability, and individualized clinical judgment.
